# Does the ICECAP-O cover the physical, mental and social functioning of older people in the UK?

**DOI:** 10.1007/s11136-018-2042-x

**Published:** 2018-11-11

**Authors:** Mariska Q. N. Hackert, Job van Exel, Werner B. F. Brouwer

**Affiliations:** 1 Erasmus School of Health Policy & Management, Erasmus University Rotterdam, Burgemeester Oudlaan 50, P.O. Box 1738, 3000 DR Rotterdam, The Netherlands; 2 Erasmus School of Economics, Erasmus University Rotterdam, Rotterdam, The Netherlands

**Keywords:** Patient-reported outcomes, Validity, Wellbeing, Health, Quality of life, United Kingdom

## Abstract

**Purpose:**

The ICEpop CAPability measure for Older people (ICECAP-O) is intended for use in economic evaluations of care services for older people. Although studies support the validity of the ICECAP-O, it does not directly ask older people about their health. This raises questions about its ability to capture health indirectly. Previous studies found mixed results in this aspect, especially for physical health. This study further investigates whether the ICECAP-O indirectly includes health.

**Methods:**

Using a cross-sectional design, a sampling agency retrieved data from 516 people aged 70 and older from the UK through an online questionnaire. The overlap in underlying constructs of the ICECAP-O and EQ-5D-5L was assessed using exploratory factor analysis. Spearman correlations and variance analysis were conducted by relating the ICECAP-O to measures of physical, mental and social functioning.

**Results:**

The ICECAP-O and EQ-5D-5L items loaded on two factors. Their overlap was limited, as four out of five EQ-5D-5L items loaded on the first factor, while four out of five ICECAP-O items loaded on the second. The ICECAP-O correlated highly with (mental and social functioning) health measures, and was able to differentiate between individuals with different scores on these measures. However, the correlation with the Barthel Index, a measure of physical functioning, was moderate.

**Conclusions:**

The ICECAP-O may not fully cover all aspects of health. Therefore, a complementary health measure should be used in addition to the ICECAP-O to capture the full benefits of care interventions for older people in economic evaluations.

## Introduction

Economic evaluations of care services for older people are indispensable. Due to population ageing, services need to be compared in terms of their costs and benefits to ensure an efficient allocation of finite resources. In economic evaluation studies, benefits of care interventions are often assessed using quality-adjusted life years (QALYs). These comprise individuals’ life duration corrected for their health during those years. Health is typically measured by multi-attribute utility instruments such as the EuroQol five-dimensional questionnaire (EQ-5D). Based on individuals’ functional abilities in various health domains (e.g. mobility, self-care and anxiety), this measure values individuals’ health on a scale from 0, being dead, to 1, being in perfect health, and negative values accounting for health states worse than being dead. By determining the QALY gain and the incremental costs of an intervention relative to a relevant comparator, those care services can be detected that yield the most health per invested monetary unit [1, 2].

However, using QALYs to calculate benefits of care services may not always be appropriate nor lead to an efficient spending of limited care resources. This holds especially when health measures like the EQ-5D do not adequately capture all relevant outcomes of the intervention under study. Services for older people often do not only intend to improve health, but also, or perhaps especially, aim to affect broader wellbeing. This may include gains in self-management, social relations or enjoyment, which are valued by older people and hence should be taken into account when assessing the benefits of an intervention [3, 4]. Failing to do so may lead decision makers to be misinformed about the full consequences of care services. Interventions may then potentially be under- or overvalued. Consequently, this may lead to the suboptimal allocation of scarce care resources [2, 5–7].

Compared to conventional health measures such as the EQ-5D, a more complete evaluation of the benefits of services for older people may be established by the ICEpop CAPability measure for Older people (ICECAP-O) [8]. Developed by extensive qualitative research in the United Kingdom (UK), the ICECAP-O focuses more broadly on quality of life rather than solely health to capture capability wellbeing among older people. Capability wellbeing focuses on individuals’ ability to achieve certain wellbeing states, irrespectively of actually doing so. The ICECAP-O measures wellbeing in five domains (using one item per domain), which are weighted to reflect their relative importance [9]. To date, validation studies show that the ICECAP-O in general correlates moderately to highly with other health and wellbeing measures and has sufficient power to differentiate between subgroups of older people [10–15].

Notwithstanding these promising validity outcomes, some caution is warranted when using the ICECAP-O in economic evaluations. Even though the ICECAP-O is considered a measure of capability wellbeing, it would be expected to cover health as one of the main pillars of overall wellbeing [2, 5, 6]. However, the ICECAP-O does not directly ask older people about their health [8]. A number of studies examined if the ICECAP-O is able to indirectly capture health through its items. Using exploratory factor analysis (EFA), Davis et al. [16] demonstrated that the items of the ICECAP-O and EQ-5D mainly capture separate factors. Based on this finding, they concluded that the items of the ICECAP-O do not adequately cover physical functioning. Their conclusion is in line with the study outcomes of Keeley et al. [17], who performed EFA on a sister measure of the ICECAP-O (i.e. the ICECAP-A aimed at the adult population). Similar findings were reported by Leeuwen et al. [14] and Hackert et al. [15], who found that the ICECAP-O correlates highly with mental functioning, but only moderately with physical functioning. Contrary to these findings, Makai et al. [12] reported a high correlation of the ICECAP-O with physical functioning. Moreover, Makai et al. [11] displayed fairly similar correlations of the ICECAP-O with mental and physical functioning. Due to these mixed findings, the relationship between ICECAP-O and health remains unclear and requires further study. After all, if the aim of an economic evaluation is to capture the full benefit of an intervention for older people, it is important to know which aspects of health are not adequately covered by the ICECAP-O and should be captured using complementary measures of health; or, when a general measure of health like the EQ-5D is used alongside the ICECAP-O, whether this would potentially lead to the double-counting of some health effects. Therefore, the extent to which the ICECAP-O includes (physical) health can have consequences both for research as well as for subsequent decision making and may make both the separate use of the ICECAP-O as well as its combined use with measures like the EQ-5D less straightforward.

Hence, this study aims to further disentangle the relation of the ICECAP-O with health. Using cross-sectional data from the UK, EFA was performed to assess whether the ICECAP-O and EQ-5D cover similar or distinct theoretical constructs and to determine whether the ICECAP-O may be used as a single comprehensive outcome measure in economic evaluations of care services for older people. Using the broad definition of health by the World Health Organization [18], convergent and discriminant validity tests were conducted to examine the relation of the ICECAP-O with the health aspects physical, mental and social functioning. Spearman rank correlations and variance analysis were used to investigate the ability of the ICECAP-O to differentiate between individuals based on e.g. their health status.

## Data and methodology

### Sampling strategy

In April–May 2015, data were obtained from 516 British people aged 70 and above using a web-based questionnaire. The age threshold of 70 years was set based on the increasing age which qualifies for senior status, as the life expectancy and the retirement age keep rising. A sampling agency was instructed to gather a representative sample in terms of age, gender and education, but representativeness proved to be difficult in relation to the selection criteria and the online recruitment strategy. Informed consent was obtained from all respondents included in the study. Participation could be terminated at any point. The minimum response time was set to 5 min based on a pilot study in which individuals were asked to properly fill out the questionnaire as quickly as possible. Because no respondent had a completion time below this threshold and all questions were mandatory, no missing data were reported.

### Measures

The *ICECAP-O* [8] includes five items of wellbeing: ‘attachment’ (love and friendship), ‘security’ (thinking about the future without concern), ‘role’ (doing things that make you feel valued), ‘enjoyment’ (enjoyment and pleasure) and ‘control’ (independence). Older people can indicate on each item to what extent they can achieve these wellbeing states using four response levels: all, a lot, a little and none. Population values were applied to weigh all items to their relative importance [9] and obtain an overall score ranging from 0 to 1, with higher scores indicating greater wellbeing.

Information was collected on respondents’ *age, gender, education* and *income*. Also, comorbidity was measured by the *Charlson comorbidity index* [19]. *Wellbeing* was measured using the reliable and valid Older People’s Quality of Life questionnaire-13 (OPQOL-13) [20], the Satisfaction With Life Scale (SWLS) [21] and Cantril’s Ladder [22]. The *OPQOL-13* [20] consists of 13 health and broader quality of life statements on which respondents can indicate their level of agreement using a 5-point response scale. Summing the responses to the statements leads to a score ranging from 13 to 65, with higher scores indicating greater wellbeing. The *SWLS* [21] uses five items to measure individuals’ level of life satisfaction. Respondents use a 7-point response scale to indicate their level of agreement with each item. A score ranging from 5 to 35 can be calculated by summing the item scores, with higher scores indicating greater satisfaction with life as a whole. *Cantril’s Ladder* [22] comprises a visual analogue scale (VAS) in the shape of a ladder ranging from 0 (worst possible life) at the bottom to 10 (best possible life) at the top, on which participants can indicate how they perceive their life.

Happiness was assessed using the Subjective Happiness Scale (SHS) [23] and Happiness VAS. The *SHS* [23] is a valid and reliable measure that consists of four items on which individuals need to rate their happiness relative to others using seven response levels. By averaging responses to the four items a score ranging from 1 to 7 was retrieved, with higher scores indicating greater happiness. The *Happiness VAS* comprised a horizontal bar on which individuals could indicate their level of happiness. The bar ranged from 0 to 10, with a 10 indicating the highest level of happiness.

Health was measured using the reliable and valid EuroQol five-dimensional five-levels questionnaire (EQ-5D-5L) [24], EuroQol Visual Analogue Scale (EQ-VAS) [24], Barthel Index [25], Geriatric Depression Scale-15 (GDS-15) [26, 27] and Brief Loneliness Scale (BLS) [28]. The *EQ-5D-5L* [24] assesses generic health using five items: ‘mobility’, ‘self-care’, ‘usual activities’, ‘pain and discomfort’ and ‘anxiety and depression’. On each item, respondents can choose between five response options to indicate how many problems they experience. A utility score was derived by weighting the relative importance of the items of the EQ-5D-5L using the value-set for England [29]. This score ranges from − 0.281 to 1, with higher scores indicating greater health and negative scores accounting for health states worse than being dead. We also performed our analyses with an unweighted index, that contained the summed responses to the five EQ-5D-5L items, ranging from 0 (i.e. perfect health) to 20 (i.e. extreme health problems on each item). This removed the influence of the value-set, which has recently been debated [30]. As our results proved to be robust for both scenarios, only the analyses with the utility score are reported. The *EQ-VAS* [24] comprises a VAS on which individuals’ can indicate their level of health. The scale ranges from 0 (worst imaginable health) to 100 (best imaginable health). To capture the diverse elements included in the broad definition of health by the WHO [18], the *Barthel Index* [25] was used to examine respondents’ *physical functioning*. The measure contains ten items on which people can indicate their ability to perform activities of daily living. Applying a scoring system leads to a score ranging from 0 to 20, with higher scores indicating greater physical functioning. The *GDS-15* [26] was used to assess participants’ *mental functioning*. The measure consists of 15 items on which individuals can indicate whether they experienced depressive symptoms in the past week. By summing the item scores a 0 to 15 scale was calculated, with higher scores indicating less mental functioning. The *BLS* [28] was used to measure individuals’ *social functioning*. The measure contains three items on which individuals can indicate their perception of social isolation using three response options. By summing the item scores a score between 3 and 9 is obtained, with higher scores indicating less social functioning.

To facilitate comparison between the measures within the context of this study, the scores of all variables, except the ICECAP-O and EQ-5D-5L, were linearly rescaled to a range between 0 and 1. For comparison with the literature, the original scores are also presented in Table 1 between brackets.

**Table 1:** Descriptive statistics of the study sample and discriminatory power of the ICECAP-O (*N* = 516)

Variable	Descriptive statistics				Discriminatory power		
	Mean	SD	Min	Max	Category	Percentage	Mean ICECAP-O
ICECAP-O	0.81	0.15	0.25	1.00			
Age	75.08	4.97	70.00	96.00	≤ 75.08	62.02	0.81
					> 75.08	37.98	0.81
Gender					Female	46.32	0.81
					Male	53.68	0.82
Education					None or primary	11.05	0.78
					Secondary school	39.53	0.82
					Further education college or university	49.42	0.81
Household income					≤ £1599-gross per month	54.26	0.79***
					> £1599-gross per month	45.74	0.84
Making ends meet					With great difficulty	4.26	0.72***
					With some difficulty	26.16	0.77
					Fairly easy	42.25	0.83
					Easily	27.33	0.84
Comorbidity	1.46	1.24	0.00	7.00	≤ 1.46	59.69	0.84***
					> 1.46	40.31	0.77
OPQOL-13^a^	0.76	0.14	0.31	1.00	≤ 0.76	55.23	0.75***
	(52.32)	(7.35)	(29.00)	(65.00)	> 0.76	44.77	0.90
SWLS^a^	0.63	0.22	0.00	1.00	≤ 0.63	41.28	0.70***
	(23.97)	(6.68)	(5.00)	(35.00)	> 0.63	58.72	0.89
Cantril’s Ladder^a^	0.70	0.19	0.00	1.00	≤ 0.70	36.24	0.70***
	(6.95)	(1.91)	(0.00)	(10.00)	> 0.70	63.76	0.88
SHS^a^	0.70	0.21	0.00	1.00	≤ 0.70	44.38	0.74***
	(5.20)	(1.24)	(1.00)	(7.00)	> 0.70	55.62	0.87
Happiness VAS^a^	0.74	0.18	0.00	1.00	≤ 0.74	38.95	0.71***
	(7.42)	(1.82)	(0.00)	(10.00)	> 0.74	61.05	0.88
EQ-5D-5L	0.74	0.24	− 0.23	1.00	≤ 0.74	32.75	0.69***
					> 0.74	67.25	0.87
EQ-VAS^a^	0.66	0.21	0.00	1.00	≤ 0.66	38.37	0.72***
	(66.44)	(20.78)	(0.00)	(100.00)	> 0.66	61.63	0.87
Barthel Index^a^	0.90	0.15	0.05	1.00	≤ 0.90	27.33	0.71***
	(17.99)	(3.08)	(1.00)	(20.00)	> 0.90	72.67	0.85
GDS-15^a^	0.26	0.24	0.00	1.00	≤ 0.26	58.33	0.89***
	(3.94)	(3.62)	(0.00)	(15.00)	> 0.26	41.67	0.71
BLS^a^	0.28	0.29	0.00	1.00	≤ 0.28	54.65	0.88***
	(4.66)	(1.72)	(3.00)	(9.00)	> 0.28	45.35	0.74

*ICECAP-O* ICEpop CAPability measure for Older people, *OPQOL-13* Older People’s Quality of Life questionnaire-13, *SWLS* Satisfaction With Life Scale, *SHS* Subjective Happiness Scale, *Happiness VAS* Happiness Visual Analogue Scale, *EQ-5D-5L* EuroQol five-dimensional five-levels questionnaire, *EQ-VAS* EuroQol Visual Analogue Scale, *GDS-15* Geriatric Depression Scale-15, *BLS* Brief Loneliness Scale

***p < 0.001, **p < 0.005, *p < 0.05

^a^Linearly rescaled scores (of 0–1); original scores of measures between brackets

### Analytic strategy

Data were analysed using the R-package (R Foundation for Statistical Computing, Vienna, Austria). Spearman rank correlations were calculated to examine the relation between the items of the ICECAP-O and EQ-5D-5L. The guidelines of Hopkins [31] were applied to evaluate their strength: < 0.10 trivial; 0.10–0.29 small; 0.30–0.49 moderate, 0.50–0.69 high; 0.70–0.89 very high; ≥ 0.90 (nearly) perfect. To determine whether both measures partly capture the same underlying constructs, as proposed in Davis et al. [16], EFA [32] was performed. EFA is a multidimensional scaling technique that uses the correlation matrix of the items of the ICECAP-O and EQ-5D-5L to reduce them to a smaller set of constructs to explore their underlying theoretical structure. Those items that belong to the same construct, also called a factor, can be interpreted to give meaning to that construct. In contrast to the sister technique Principal Component Analysis, EFA realistically assumes that a part of the variance of each item is unique, which holds that the variance of each item cannot be fully explained by the other items. The use of the method in this study was approved based on the Barlett’s test of sphericity [33], Kaiser–Meyer–Olkin measure of sampling adequacy (KMO) [33] and a multicollinearity test conducted using the package USDM [34]. Based on conventional maximum likelihood extraction and Pearson correlations [35, 36], diverse methods were used to extract the appropriate number of factors, including a scree plot, parallel analysis and a very simple structure [32, 33, 36]. Using the package PSYCH [37], multiple robustness checks were performed. Polychoric correlations were used to test the influence of the possible violation of linearity [35, 36]. Also, principal axis factoring was used to check the impact of the possible violation of multivariate normality [38]. While interpreting the models, Oblimin rotation was applied using the package GPArotation [39] to allow factors to be correlated. Because the various models displayed minor differences, only the EFA based on maximum likelihood and Pearson correlations is presented, as this model gives insight in the uniqueness of all items. Only the highest factor loading for each item was reported, which needed to be equal or greater than 0.40 to be considered reliable for interpretation [40]. Next, convergent validity was tested by calculating Spearman rank correlations. The ICECAP-O was related to various wellbeing (OPQOL-13, SWLS and Cantril’s Ladder), happiness (SHS and Happiness VAS) and health (EQ-5D-5L, EQ-VAS, Barthel Index, GDS-15 and BLS) measures. Using the package PPCOR [41] Spearman rank correlations were checked, controlling for individuals’ age, gender, education and income (all included as interval or ratio measures). Discriminant validity was tested by the ability of the ICECAP-O to differentiate between subgroups of older people, which were created using the background characteristics and previously mentioned measures. In combination with the Levene’s test on the violation of the homogeneity of variances, *T* tests were performed for two group comparisons and one-way ANOVA’s for multiple group comparisons.

## Results

### Sample characteristics

The descriptive statistics of the study sample are displayed in Table 1. The respondents were on average 75 years old. A slight majority was male and 49% completed at least further education college. About 46% had an income of at least £1600/month, whereas 70% reported to make ends meet (fairly) easily. The mean comorbidity score was 1.46 with a range between 0 and 7 diseases mentioned. 23% reported no diseases, whereas 37% reported one disease and 40% reported two diseases or more.

**Fig. 1:**
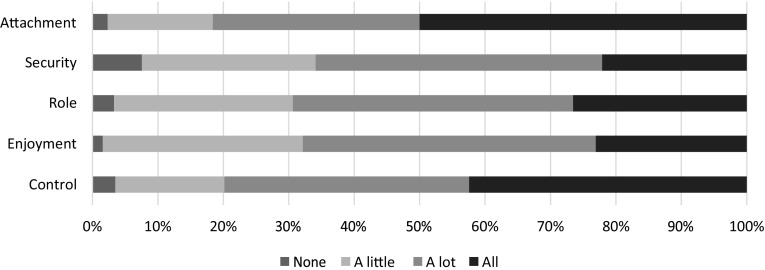
Response distribution on the items of the ICECAP-O (*N* = 516). *ICECAP-O* ICEpop CAPability measure for Older people

The mean overall score on the ICECAP-O was high (0.81). Most of the older people reported a great level of wellbeing on all ICECAP-O items (see Fig. 1). The lowest levels were mentioned on the items ‘security’ and ‘enjoyment’. In contrast, wellbeing measured by the OPQOL-13, SWLS and Cantril’s Ladder was moderate to high, with mean values of 0.76, 0.63 and 0.70.

Furthermore, older people reported a high level of happiness, with a mean score of 0.70 on the SHS and 0.74 on the Happiness VAS. Comparable levels were derived for health, measured by the EQ-5D-5L (0.74) and EQ-VAS (0.66). On average, the respondents were able to function independently and had only minor signs of depression and social isolation, as evident from the mean values of the Barthel Index (0.90), GDS-15 (0.26) and BLS (0.28).

### Dimensionality

**Fig. 2:**
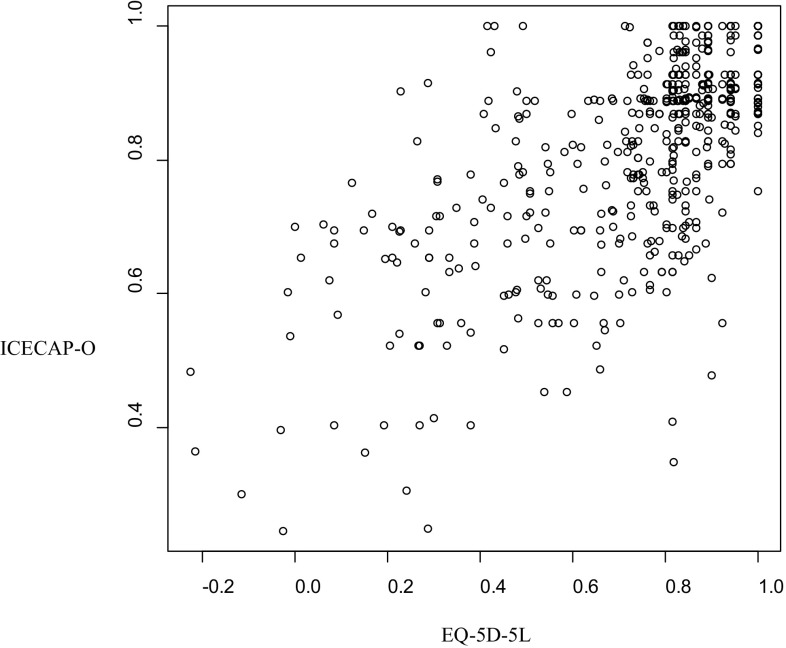
Scatterplot of the overall scores of the ICECAP-O and EQ-5D-5L (*N* = 516). *ICECAP-O* ICEpop CAPability measure for Older people, *EQ-5D-5L* EuroQol five-dimensional five-levels questionnaire

In Fig. 2, the association between the ICECAP-O and EQ-5D-5L is displayed in a scatterplot. Spearman rank correlations between the overall scores and items of both measures are displayed in Table 2, all controlled for age, gender, education and income, and original bivariate correlations are presented between brackets.

**Table 2:** Spearman correlation matrix of the items and overall scores of the ICECAP-O and EQ-5D-5L (*N* = 516)

ICECAP-O	EQ-5D-5L					
	Overall score	Items				
		Mobility	Self-care	Usual activities	Pain/discomfort	Anxiety/depression
Overall score	0.63***	0.49***	0.43***	0.55***	0.38***	0.53***
	(0.65***)	(0.50***)	(0.45***)	(0.57***)	(0.42***)	(0.55***)
Items						
Attachment	0.25***	0.15***	0.15***	0.15***	0.10*	0.30***
	(0.26***)	(0.15***)	(0.15***)	(0.16***)	(0.13**)	(0.32***)
Security	0.43***	0.27***	0.25***	0.35***	0.26***	0.45***
	(0.46***)	(0.30***)	(0.27***)	(0.38***)	(0.30***)	(0.47***)
Role	0.56***	0.47***	0.40***	0.51***	0.33***	0.40***
	(0.58***)	(0.49***)	(0.42***)	(0.53***)	(0.37***)	(0.42***)
Enjoyment	0.52***	0.40***	0.31***	0.43***	0.32***	0.44***
	(0.55***)	(0.43***)	(0.34***)	(0.46***)	(0.36***)	(0.46***)
Control	0.62***	0.58***	0.54***	0.63***	0.44***	0.35***
	(0.65***)	(0.59***)	(0.55***)	(0.64***)	(0.47***)	(0.37***)

Controlled for age, gender, educational level and income; original correlation coefficients between brackets

*ICECAP-O* ICEpop CAPability measure for Older people, *EQ-5D-5L* EuroQol five-dimensional five-levels questionnaire

***p < 0.001, **p < 0.005, *p < 0.05

The overall score of the ICECAP-O correlated highly with the overall score of the EQ-5D-5L (0.63). More specifically, the ICECAP-O items ‘role’, ‘enjoyment’ and ‘control’ correlated highly with the overall score of the EQ-5D-5L, while the items ‘attachment’ and ‘security’ showed only small to moderate correlations. In addition, the ICECAP-O items correlated at least moderately with the EQ-5D-5L items, except for the items ‘attachment’ and ‘security’.

The EFA results are presented in Table 3. When the ICECAP-O items were combined with the items of the EQ-5D-5L, the scree plot, parallel analysis and very simple structure indicated either a two or three factor structure. As the literature supports the two factor structure, we decided to display those results.

**Table 3:** Oblimin rotated factor loadings for the items of the ICECAP-O and EQ-5D-5L (N = 516)

	Uniqueness	Factors^a^	
		1	2
ICECAP-O			
Attachment	0.72		0.63
Security	0.54		0.68
Role	0.37		0.64
Enjoyment	0.36		0.78
Control	0.40	0.58	
EQ-5D-5L			
Mobility	0.24	0.90	
Self-care	0.42	0.76	
Usual activities	0.19	0.89	
Pain/discomfort	0.49	0.71	
Anxiety/depression	0.65		0.55
Proportion of total variance		0.31	0.23
Correlation with factor 1			0.59

*ICECAP-O* ICEpop CAPability measure for Older people, *EQ-5D-5L* EuroQol five-dimensional five-levels questionnaire

^a^Only presented is the highest factor loading per item, which is also ≥ 0.40

The first factor included the ICECAP-O item ‘control’ and the EQ-5D-5L items ‘mobility’, ‘self-care’, ‘usual activities’ and ‘pain and discomfort’. Davis et al. [16] labelled this factor as ‘physical functioning’. All items had a low uniqueness (≤ 0.49) and together, they explained 31% of the total variance of all items. Factor two comprised the ICECAP-O items ‘attachment’, ‘security’, ‘role’ and ‘enjoyment’ and the EQ-5D-5L item ‘anxiety and depression’. Davis et al. [16] labelled this factor as ‘psychosocial wellbeing’. In total, they explained 23% of the variance of all items. In particular, the items ‘attachment’, ‘security’ and ‘anxiety and depression’ showed high unique variances (≥ 0.54). The overlap between the items of the ICECAP-O and EQ-5D-5L was even less in the three factor structure, where the ICECAP-O item ‘control’ loaded on a third factor.

### Convergent validity

Table 4 demonstrates the Spearman rank correlations of the overall score of the ICECAP-O with the wellbeing, happiness and health measures.

**Table 4:** Spearman correlation matrix of the items and overall score of the ICECAP-O with diverse wellbeing (OPQOL-13—Cantril’s Ladder), happiness (SHS—Happiness VAS) and health (EQ-5D-5L—BLS) measures (*N* = 516)

ICECAP-O	OPQOL-13	SWLS	Cantril’s Ladder	SHS	Happiness VAS	EQ-5D-5L	EQ-VAS	Barthel Index	GDS-15	BLS
Overall score	0.66***	0.72***	0.67***	0.56***	0.61***	0.63***	0.58***	0.39***	− 0.64***	− 0.55***
	(0.68***)	(0.74***)	(0.69***)	(0.57***)	(0.63***)	(0.65***)	(0.60***)	(0.42***)	(− 0.66***)	(− 0.57***)
Items										
Attachment	0.37***	0.47***	0.40***	0.38***	0.37***	0.25***	0.25***	0.07	− 0.32***	− 0.51***
	(0.37***)	(0.47***)	(0.40***)	(0.37***)	(0.38***)	(0.26***)	(0.26***)	(0.07)	(− 0.32***)	(− 0.51***)
Security	0.47***	0.57***	0.48***	0.42***	0.47***	0.43***	0.40***	0.21***	− 0.47***	− 0.36***
	(0.50***)	(0.59***)	(0.50***)	(0.43***)	(0.48***)	(0.46***)	(0.42***)	(0.23***)	(− 0.49***)	(− 0.38***)
Role	0.56***	0.55***	0.55***	0.45***	0.51***	0.56***	0.51***	0.38***	− 0.52***	− 0.44***
	(0.59***)	(0.58***)	(0.58***)	(0.47***)	(0.53***)	(0.58***)	(0.54***)	(0.40***)	(− 0.55***)	(− 0.46***)
Enjoyment	0.58***	0.61***	0.60***	0.50***	0.56***	0.52***	0.49***	0.30***	− 0.55***	− 0.50***
	(0.61***)	(0.64***)	(0.63***)	(0.52***)	(0.58***)	(0.55***)	(0.53***)	(0.34***)	(− 0.58***)	(− 0.52***)
Control	0.52***	0.48***	0.50***	0.37***	0.41***	0.62***	0.55***	0.49***	− 0.49***	− 0.31***
	(0.55***)	(0.51***)	(0.52***)	(0.39***)	(0.42***)	(0.65***)	(0.58***)	(0.50***)	(− 0.52***)	(− 0.34***)

Controlled for age, gender, educational level and income; original correlation coefficients between brackets

*ICECAP-O* ICEpop CAPability measure for Older people, *OPQOL-13* Older People’s Quality of Life questionnaire-13, *SWLS* Satisfaction With Life Scale, *SHS* Subjective Happiness Scale, *Happiness VAS* Happiness Visual Analogue Scale, *EQ-5D-5L* EuroQol five-dimensional five-levels questionnaire, *EQ-VAS* EuroQol Visual Analogue Scale, *GDS-15* Geriatric Depression Scale-15, *BLS* Brief Loneliness Scale

***p < 0.001, **p < 0.005, *p < 0.05

The ICECAP-O correlated (very) highly with the wellbeing measures OPQOL-13, SWLS and Cantril’s Ladder. Comparable correlations were found with the happiness (SHS and Happiness VAS) and health (EQ-5D-5L and EQ-VAS) measures. The ICECAP-O correlated highly with the mental and social functioning measures GDS-15 and BLS. In contrast, its correlation with the physical health measure Barthel Index was only moderate. The correlations of the items of the ICECAP-O with previous measures showed a similar picture, although the correlations were generally lower.

### Discriminant validity

In Table 1, the discriminatory power of the ICECAP-O is presented. The overall score of the ICECAP-O differentiated between older people based on their income. The group with a higher income or who made ends meet (fairly) easily had a greater mean ICECAP-O score than those who did not reach these levels. Also, the ICECAP-O discriminated between respondents based on their score on the wellbeing (OPQOL-13, SWLS and Cantril’s Ladder), happiness (SHS and Happiness VAS) and health (EQ-5D-5L, EQ-VAS, Barthel Index, GDS-15 and BLS) measures. Those who scored equal or below the average value differentiated from those who scored above this threshold.

## Discussion

### Main findings

In a sample of 516 adults aged 70 years and older from the UK, EFA showed that the ICECAP-O and the health measure EQ-5D-5L tap into two shared underlying constructs. Nevertheless, the overlap between both measures was limited, as four out of five EQ-5D-5L items loaded on the first factor, while four out of five ICECAP-O items loaded on the second. Using Spearman rank correlations and variance analysis in combination with a broad range of measures covering the definition of health by the WHO [18], the convergent and discriminant validity of the ICECAP-O was examined. The ICECAP-O correlated highly with mental (GDS-15) and social (BLS) functioning and overall measures of health (EQ-5D-5L, EQ-VAS), but only a moderate correlation was found with the Barthel Index, a measure of physical functioning. Finally, the ICECAP-O correlated highly with wellbeing (OPQOL-13, SWLS and Cantril’s Ladder) and happiness (SHS and Happiness VAS) measures, and was able to differentiate between subgroups of older people based on their income and their scores on the above mentioned measures.

### Comparability with previous studies

This study confirmed that the ICECAP-O largely covers health with the exception of physical functioning. The factor structure of the items of the ICECAP-O and EQ-5D-5L was similar to that obtained by Davis et al. [16] and Keeley et al. [17], although the ICECAP-O item ‘control’ and the EQ-5D-5L item ‘pain and discomfort’ loaded on a unique factor in this study. The Spearman rank correlations seem to support this conclusion. In contrast to the studies of Makai et al. [11, 12], but in line with Leeuwen et al. [14] and Hackert et al. [15], the ICECAP-O correlated to a greater extent with the GDS-15, a measure of mental functioning, than the Barthel Index, a measure of physical functioning. Also in contrast to Makai et al. [11], the ICECAP-O correlated highly with the social functioning measure BLS. Moreover, the ICECAP-O correlated highly with the generic health measures EQ-5D-5L and EQ-VAS, and diverse wellbeing (OPQOL-13, SWLS and Cantril’s Ladder) and happiness (SHS and Happiness VAS) measures. Although the strength of these correlations varied to some extent, this study broadly confirmed the validity outcomes of previous studies performed in the Netherlands [11, 14], UK [15], Germany [12] and Australia [10]. Finally, the research findings supported the discriminatory validity outcomes obtained in previous studies [11–13, 15]. The ICECAP-O differentiated between older people based on their income, and their score on the measures used to check the convergent validity.

### Study limitations and strengths

Some study limitations are worth mentioning. The results of this study are limited by its cross-sectional design, as we were unable to examine the extent to which the ICECAP-O is sensitive to the impact of various care services and changes in (self-perceived) wellbeing and health. The generalizability of the study outcomes may be hampered by the use of an online panel. Due to the limited online participation of the eldest and the lower educated, they were underrepresented in this study. We could not access data of respondents who did not complete the questionnaire. We acknowledge that this limits our insight in possible issues regarding the response rate and completion of the measures. Nevertheless, respondents showed considerable heterogeneity, and the study included a wide range of measures to test the validity of the ICECAP-O. Repeating all analyses in a dataset excluding respondents with a response time below 10 or above 60 min did not affect our main results.

### Research and policy recommendations

The ICECAP-O may be a promising candidate to replace health measures in evaluation studies of care for older people. The ICECAP-O not only includes specific items on wellbeing, but is also able to capture aspects of health through them. However, further research is required on several aspects of the measure. So far, analyses on the coverage of similar underlying factors as the EQ-5D were conducted in Canada [16] and the UK. Research in other countries and in diverse subgroups of older people should be stimulated to re-examine the uptake of physical functioning by the ICECAP-O. Whether or not the ICECAP-O and physical functioning are related to each other may depend on the sample under study. Limited overlap in the factor structure and moderate correlations (0.30–0.49) between the ICECAP-O and physical functioning were observed in samples of frail older people and social care users [14–16]. On the other hand, high correlations were reported in a sample of post-hospitalized older people (− 0 .51) [11] and a sample of older people diagnosed with dementia (0.72) [12]. In particular in the latter study the correlation between the ICECAP-O and physical functioning was very high, but it needs noting that nursing professionals were used as proxy respondents in this study, which may have affected this outcome. More knowledge on the relationship between the ICECAP-O and physical functioning in different samples remains warranted. To strengthen the evidence in this area, future studies could relate the ICECAP-O to clinical measures of physical and mental health in addition to the self-reported questionnaires used here and in other studies. If future studies support the results discussed here, efforts should be made to integrate this aspect of health into the measure, as using the ICECAP-O and a generic measure of health like EQ-5D simultaneously may lead to the double-counting of certain health effects. Also, population values should be developed in other countries to determine the relative importance of the ICECAP-O items there. Finally, the ICECAP-O should be examined on its sensitivity-to-change. The Minimal Clinical Important Difference (MCID) should be calculated to derive the smallest change in the ICECAP-O that is meaningful for the elderly. Using the one-half standard deviation benchmark, in this study the MCID would be 0.08.

## Conclusion

This study supported the validity outcomes of the ICECAP-O found in previous studies [10–16]. The ICECAP-O displayed convergent and discriminant validity with diverse wellbeing, happiness and health measures. However, physical functioning did not appear to be fully captured, as most of the ICECAP-O and EQ-5D-5L items loaded on different factors in the EFA, and the ICECAP-O correlated moderately with the Barthel Index. As the ICECAP-O apparently does not fully cover the health effects of interventions, the measure should be used with caution and perhaps in addition to a complementary measure of health to evaluate the full benefits of interventions for older people. How both measures can be combined should be investigated further.
